# Differential impacts of audiovisual information on empathic accuracy in people with schizophrenia and high social anhedonia

**DOI:** 10.1017/S003329172610364X

**Published:** 2026-03-23

**Authors:** Miao Wang, Guo-Hui Zhu, Juan Yang, Xin-Wei Fu, Li-Ying Zhang, Ding-Ding Hu, Simon Lui, Yan-Yu Wang, Yi Wang, Raymond C.K. Chan

**Affiliations:** 1 Neuropsychology and Applied Cognitive Neuroscience Laboratory, State Key Laboratory of Cognitive Science and Mental Health, Institute of Psychology Chinese Academy of Sciences, Beijing, China; 2 Department of Psychology, University of Chinese Academy of Sciences, Beijing, China; 3 School of Psychology, Shandong Second Medical University, Weifang, Shandong, China; 4 Weifang Mental Health Centre, Weifang, Shandong, China; 5 Department of Psychiatry, School of Clinical Medicine, The University of Hong Kong, Hong Kong Special Administrative Region, China

**Keywords:** audiovisual information, avatar, empathic accuracy, schizophrenia

## Abstract

**Background:**

Empathy involves communicating and understanding others’ emotion in multisensory contexts, including visual and auditory modalities. Schizophrenia (SCZ) patients have impaired empathy, but whether the impact of visual/auditory context would be altered in SCZ patients and people with high social anhedonia (HSoA) remained unclear.

**Methods:**

We administered the modified Chinese version of the Empathic Accuracy Task (EAT) to clinical (50 SCZ patients and 50 healthy controls) and subclinical samples (59 HSoA and 60 low social anhedonia [LSoA] participants). The EAT employed audio-only, audiovisual, and audioavatar visual conditions to assess the impact of multimodal information on empathy during positive and negative emotional events.

**Results:**

In positive-valenced context, SCZ patients performed worse than controls in cognitive and affective empathy. The Modality-by-Group interaction on empathic accuracy was significant, that is, SCZ patients performed worse than controls in both audiovisual and audioavatar visual conditions, but comparable to controls in audio-only condition. In negative-valenced context, SCZ patients performed worse than controls in cognitive empathy. The Modality-by-Group interaction on empathic accuracy was significant, that is, SCZ patients performed worse than controls in audio-only and audiovisual conditions. Moreover, HSoA participants exhibited lower cognitive empathy than controls in positive-valenced context; and lower cognitive empathy and empathic motivation in negative-valenced context. No significant Modality-by-Group interaction was found in the HSoA–LSoA sample.

**Conclusions:**

SCZ patients have generalized impairments of cognitive and affective empathy across positive and negative contexts, particularly in multimodal conditions. HSoA individuals are primarily impaired in cognitive empathy and empathic motivation.

## Introduction

Although antipsychotics can alleviate positive symptoms, many schizophrenia (SCZ) patients suffer from negative symptoms and functional impairments (Couture, Penn, & Roberts, 2006; Halverson et al., 2019). SCZ patients also have markedly elevated risk of suicide, compared to the general populations (Bertuccio et al., 2024). Empathy refers to the ability to understand and feel others’ emotional states (Preston & de Waal, 2002). Cognitive and affective empathy are impaired in patients with SCZ (Bonfils, Lysaker, Minor, & Salyers, 2016, 2017), undermining patients’ everyday social interactions (Green, Horan, & Lee, 2015; Vaskinn et al., 2018). Moreover, impaired empathy is associated with suicidal behavior and risk of bullying by others (Chu, Lu, & Huang, 2025; Serafini et al., 2023; van Noorden, Haselager, Cillessen, & Bukowski, 2015; Wang et al., 2020a). Multisensory processing (mostly visual and auditory modalities) is the primary channel for social perception and empathic accuracy (Gesn & Ickes, 1999; Hall & Schmid Mast, 2007; Jospe et al., 2020; Kraus, 2017; Ong et al., 2023). Emotion recognition is a core process of empathy (Derntl et al., 2009) and is impaired in SCZ patients who are presented with audio-only and audiovisual information (Giannitelli et al., 2015; Thaler et al., 2013). Vogel et al. (2016) found SCZ patients having relatively preserved emotion recognition in audio-only condition, suggesting that the impaired emotion recognition might emerge primarily from altered processing in audiovisual condition. However, Simpson, Pinkham, Kelsven and Sasson (2013) found that SCZ patients showed poorer emotion recognition in audio-only and visual-only conditions, rather than audiovisual condition. Indeed, visual and auditory information exert differential effects on the recognition of positive versus negative emotions (Paulmann & Pell, 2011; Zhang et al., 2018). For instance, positive emotions tend to be more accurately recognized under visual (e.g. facial expressions) instead of auditory (e.g. vocal tones) conditions, whereas negative emotions are recognized comparably well in the two modalities (Zhang et al., 2018).

SCZ patients have impaired cognitive empathy, regardless of the different types of tasks, including paradigms involving visual stimuli (i.e. Berger et al., 2019; Kamp et al., 2025; Karpouzian-Rogers et al., 2021; Smith et al., 2014), auditory stimuli (i.e. Atoui et al., 2018), and audiovisual stimuli (i.e. Baez et al., 2013; de Jong et al., 2018; Harenski et al., 2017; van Donkersgoed et al., 2019). Using the Empathic Accuracy Task (EAT), some studies found that intensity of emotional expression in the stimuli could differentially affect empathy in SCZ patients, that is, they performed well in low-expressive stimuli, but poorly in high-emotional expressions (Harvey et al., 2013; Lee et al., 2011). Studies using visual information tasks showed that SCZ patients had poorer affective empathy than controls (i.e. Benedetti et al., 2009; Derntl et al., 2009; Lee et al., 2010, Smith et al., 2014); but the two groups showed similar affective empathy in tasks using silent or short audiovisual video clips (Knobloch et al., 2024; Ramos-Loyo, Mora-Reynoso, Sánchez-Loyo, & Medina-Hernández, 2012).

Schizotypy is a latent personality organization reflecting vulnerability to schizophrenia (Meehl, 1962). Physical and social anhedonia are negative dimension of schizotypy (Wang, Lui, & Chan, 2024). In particular, high social anhedonia (HSoA) constitutes a high-risk condition for developing SCZ (Kwapil, 1998) and is associated with attenuated empathy deficits relative to SCZ (Dominguez et al., 2011). Previous studies found a significant negative correlation between social anhedonia and empathy (Wang et al., 2020b). HSoA individuals exhibited poorer empathy than people with low social anhedonia (Gu et al., 2025; Guo et al., 2023; Pflum & Gooding, 2018). Research using samples with HSoA can circumvent the confounding variables (e.g. antipsychotics) inherent in clinical patients, and provide early detection frameworks (Barrantes-Vidal, Grant, & Kwapil, 2015).

Several unresolved issues concerning empathy in SCZ spectrum disorders are notable. First, previous studies emphasized the influence of audiovisual information on empathy, but the traditional video materials were mostly real-human videos, lacking standardization and manipulation of visual information. Avatar technology (computer-generated agents) could replicate human morphology and kinematics (Boulic, Bécheiraz, Emering, & Thalmann, 1997; Sung, Han, Bae, & Kwon, 2022). These programmable virtual humans enable precise control over facial muscle dynamics and emotional expression gradients, better than real-human video materials. However, few empirical studies have investigated the difference between Avatar and real-human videos in influencing empathy. Moreover, very few studies have explored whether empathy deficits in SCZ would be related to the mode/channels (visual and auditory) for presenting materials. Furthermore, previous research on empathy in HSoA samples was limited to unimodal stimulation materials, and seldom used multimodal audiovisual materials. Finally, no study has explored the differential impacts of visual and auditory information on the empathy performance of SCZ patients and individuals with HSoA.

This study aimed to examine the impacts of visual modalities on empathy using the modified Chinese version of the EAT (Wang et al., 2025) in SCZ patients and people with HSoA. We hypothesized that SCZ patients would have significantly reduced empathic accuracy, cognitive and affective empathy relative to controls, across different conditions. We also hypothesized that empathy deficits would be inversely correlated with clinical symptoms (in particular negative symptoms) in SCZ patients. Finally, we hypothesized that people with HSoA would have attenuated deficit of empathy relative to SCZ patients, involving affective empathy.

## Methods

### Participants

This study utilized two independent samples. The SCZ–Control sample comprised 50 SCZ patients recruited from the psychiatric department of Weifang Mental Health Center, and 50 healthy controls from the neighboring community. The inclusion criteria for clinical patients were: (1) DSM-5 (American Psychiatric Association, 2013) diagnosis of SCZ, (2) aged 18–60, (3) IQ ≥70, (4) normal (or corrected-to-normal) hearing and visual acuity, and (5) clinical stabilization. The exclusion criteria for clinical patients included (1) history of brain injury, major physical illness, or organic brain lesions, (2) lifetime history of psychoactive substance dependence or abuse, (3) neurological organic diseases, and (4) history of transcranial magnetic stimulation therapy or electroconvulsive therapy in the past 14 days. The inclusion criteria for controls included (1) absence of personal and family history of psychiatric disorder as confirmed by the MINI International Neuropsychiatric Interview (M.I.N.I.) (Lecrubier et al., 1997), (2) normal (or corrected) hearing and visual acuity, and (3) IQ ≥70. The exclusion criteria for controls included (1) history of traumatic brain injury, (2) organic brain lesions, (3) psychoactive substance abuse, and (4) neurological diseases.

The HSoA–LSoA sample comprised 59 participants with HSoA and 60 participants with low social anhedonia (LSoA). The inclusion criteria for HSoA included (1) CSAS ≥17 (Zhang et al., 2020), (2) aged 18–25, and (3) normal (or corrected-to-normal) hearing and visual acuity. The inclusion criteria for LSoA included (1) CSAS <11 (Zhang et al., 2020); (2) aged 18–25, (3) absence of traumatic brain injury and neurological diseases, and (4) normal (or corrected-to-normal) hearing and visual acuity. The exclusion criteria for both HSoA and LSoA included (1) M.I.N.I.-identified psychiatric illness, and (2) lifetime history of drug dependence, substance dependence, and alcohol dependence.

This study was approved by the Ethics Committee of Shandong Second Medical University (2023YX080). All participants gave written informed consent.

### Measures

*The modified Chinese version of the EAT* (Wang et al., 2025) was used in the current study, which comprised three *Modality Conditions*: audio-only, audiovisual, and audioavatar visual condition. Six videos were randomly selected from a standardized video library (Hu et al., 2024) and assigned to each condition (one positive and one negative clips). The audiovisual condition presented the raw, unprocessed footage; the audio-only condition extracted the audio from the original video; the audioavatar visual condition were created using Reallusion® Character Creator©, Reallusion® iClone®, and Adobe® Premiere Pro® software (Wang et al., 2025). As in the original version of EAT, participants were asked to watch each video of emotional stories, and continuously rated the narrator’s emotions using a 9-point scale (1 = very negative, 9 = very positive). The ratings of Perspective Taking, the Target’s Emotional Valence and Arousal reflected the participants’ cognitive empathy; while the Emotional Contagion, self-emotional valence and arousal reflected the participants’ affective empathy. The ratings of empathic concern, help, and effort level were filled in only after watching the negative videos, and were used to measure the participants’ empathic motivation. Spearman’s correlation coefficient between the participant and the narrator’s ratings was calculated and Fisher’s *z* transformed to measure the Empathic Accuracy (EA).

In addition, the Positive and Negative Syndrome Scale (PANSS) (Kay, Fiszbein, & Opler, 1987; Si et al., 2004) and the Clinical Assessment Interview for Negative Symptoms (CAINS) (Chan et al., 2015; Kring et al., 2013) were administered to assess the clinical symptoms in SCZ patients. The Chinese version of the Wechsler Adult Intelligence Scale–Revised (WAIS-R) (Gong & Dai, 1984) was used to assess estimated IQ through four subtests, that is, General Knowledge, Arithmetic, Similarities and Digit Span. The Chapman Social Anhedonia Scale (CSAS) and the Chapman Physical Anhedonia Scale (CPAS) (Chapman, Chapman, & Raulin, 1976) had been found to have excellent psychometric properties (with Cronbach’s alpha coefficients of 0.84 and 0.86, respectively) and were used to measure the participants’ levels of anhedonia. Likewise, the Cognitive and Affective Empathy Scale (QCAE) (Liang et al., 2019; Reniers et al., 2011) demonstrated a Cronbach’s alpha coefficient of 0.88, and was used to assess the participants’ trait cognitive and affective empathy. Finally, the First Episode Social Functioning Scale (FESFS) (Lecomte et al., 2014; Wang et al., 2013) demonstrated a Cronbach’s alpha coefficient of 0.94, and was used to evaluate the participants’social functioning.

### Data analysis

We conducted independent samples *t* tests to compare the differences in demographic variables and scores on the self-report scales between the groups. The 3 (Modality: audio-only, audiovisual, audioavatar visual condition) × 2 (Group: SCZ vs. HC; or HSoA vs. LSoA) mixed models of analysis of variance (ANOVA) were performed to examine the Modality, and Group main effects, and the Modality-by-Group interaction on the EAT performance, during the positive and negative video conditions separately for each sample. Bonferroni corrections were applied for *post hoc* pairwise comparisons. Pearson’s correlation analysis was used to explore the relationship between clinical symptoms and empathy deficits in the patient group. False discovery rate (FDR)-adjusted *p* values were reported for correlation analyses. Data analyses were performed using the SPSS, with a significance level set at *p* or adjusted *p* < 0.05.

## Results

### SCZ–control sample

Table 1 shows the demographics, self-report scales, and clinical symptoms. The two groups did not differ in gender and age. However, SCZ patients had lower years of education and estimated IQ, higher levels of physical and social anhedonia, and lower scores of cognitive empathy than healthy controls.

**Table 1.** Demographics and clinical characteristics of schizophrenia–control sample

	SCZ group (*n* = 50)		Control group (*n* = 50)		*t/χ^2^*	*p*	*df*	Cohen’s *d* [95% CI]
	*M*	*SD*	*M*	*SD*				
Sex (male:female)	26:24		24:26		0.160	0.689	1	
Age (years)	37.58	9.32	36.42	9.12	0.629	0.531	98	0.13 [−0.27,0.52]
Years of education	11.10	2.74	13.90	2.84	−5.011	<0.001	98	−1.00 [−1.42,–0.58]
IQ estimates	89.26	12.19	113.62	12.40	−9.903	<0.001	98	−1.98 [−2.46,–1.50]
Duration of illness (year)	13.09	7.62						
Chlorpromazine (mg/day)	431.04	250.67						
PANSS_positive	10.28	3.46						
PANSS_negative	16.04	6.13						
PANSS_general psychopathology	25.60	4.81						
PANSS_total	51.92	10.98						
CAINS_MAP	17.54	6.83						
CAINS_EXP	5.20	3.80						
CAINS_total	22.82	9.22						
CSAS	13.70	6.56	10.10	4.55	3.191	0.002	87.26	0.64 [0.24,1.04]
CPAS	18.92	7.50	13.52	8.09	3.461	0.001	98	0.69 [0.29,1.09]
QCAE_CE	52.80	10.96	59.86	9.02	−3.493	0.001	97	−0.70 [−1.11,–0.29]
QCAE_AE	27.82	5.09	28.86	4.84	−1.037	0.302	96	−0.21 [−0.61,0.19]
SFS total	63.85	9.81	65.34	6.87	−0.865	0.389	83.84	−0.18 [−0.57,0.22]

*Note:* AE, affective empathy; CAINS, clinical assessment interview for negative symptoms; CE, cognitive empathy; CSAS, Chapman Social Anhedonia Scale; CPAS, Chapman Physical Anhedonia Scale; EXP, expression; MAP, motivation and pleasure; QCAE,  Cognitive and Affective Empathy Scale; PANSS, Positive and Negative Syndrome Scale; SFS, First Episode Social Functioning Scale.

#### EAT performance in positive-valenced videos

**Figure 1.**
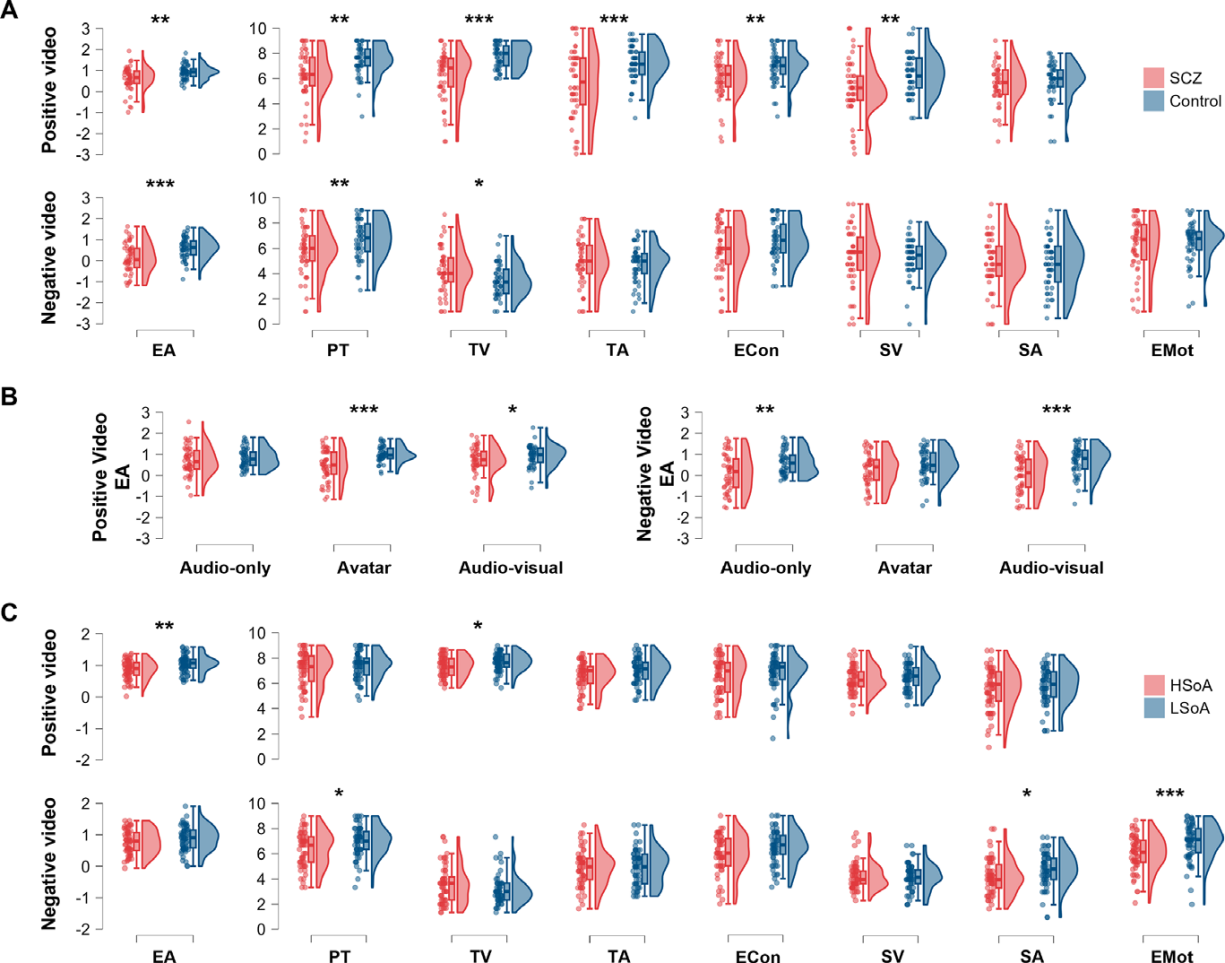
Performance on the empathic accuracy task. *Note*: Panel A illustrates the main effects of group (SCZ vs. Control); Panel B illustrates the Modality-by-Group interaction effect. Panel C illustrates the main effects of group (HSoA vs. LSoA). EA, empathic accuracy; Econ, emotional contagion; EMot, empathic motivation; HSoA, high social anhedonia; LSoA, low social anhedonia; PT, perspective taking; SA, self-emotional arousal; SCZ, schizophrenia; SV, self-emotional valence; TA, target’s emotional arousal; TV, target’s emotional valence.

As shown in Table 2 and Figure 1A, significant Group *main effect* was found, suggesting that SCZ patients scored significantly lower than controls across different cognitive empathy variables (i.e. Empathic Accuracy, Perspective Taking, Target’s Emotional Valence, and Target’s Emotional Arousal) and affective empathy variables (Emotional Contagion and Self-Emotional Valence). Moreover, we found a significant Modality *main effect* on the Target’s Emotional Valence ratings of cognitive empathy, *F*(2, 196) = 3.43, *p* = 0.034, *η*_*p*_^2^ = 0.034. Specifically, ratings in the audiovisual condition (*M* = 7.21, *SD* = 2.01) were significantly higher than those in the audioavatar visual condition (*M* = 6.72, *SD* = 2.10, Bonferroni-adjusted *p* = 0.032, Cohen’s *d* = − 0.26). We also found a significant Modality-by-Group *interaction effect* for the Empathic Accuracy, *F*(2, 196) = 5.61, *p* = 0.004, *η*_*p*_^2^ = 0.054 (see Supplementary Table S1). As shown in Figure 1B, SCZ patients showed significantly lower empathic accuracy than controls under both audiovisual (Bonferroni-adjusted *p* = 0.039, Cohen’s *d* = − 0.42) and audioavatar visual conditions (Bonferroni-adjusted *p* < 0.001, Cohen’s *d* = −0.93), whereas no significant group difference was found under audio-only condition. Within-group comparisons further indicated that, among SCZ patients, the Empathic Accuracy in audio-only condition was significantly higher than audioavatar visual condition (Bonferroni-adjusted *p* = 0.017), but no significant differences could be found among three conditions in the control participants.

**Table 2.** Group comparisons between schizophrenia patients and healthy controls on the EAT performance

		SCZ group(*n* = 50)		HC group(*n* = 50)		*F*	*p*	*df*	*η*_*p*_^2^
		*M*	*SD*	*M*	*SD*				
Positive videos	EA	0.62	0.56	0.92	0.33	11.06	0.001	1	0.101
	PT	6.30	1.91	7.42	1.23	12.12	0.001	1	0.110
	TV	6.25	1.92	7.73	0.94	23.97	<0.001	1	0.197
	TA	5.95	1.90	7.05	1.00	13.09	<0.001	1	0.118
	Econ	6.09	1.75	6.99	1.31	8.30	0.005	1	0.078
	SV	5.67	1.60	6.43	1.11	7.58	0.007	1	0.072
	SA	5.61	1.54	5.77	1.52	0.30	0.587	1	0.003
Negative videos	EA	0.16	0.69	0.61	0.50	14.16	<0.001	1	0.126
	PT	5.78	1.86	6.74	1.52	7.97	0.006	1	0.075
	TV	4.25	1.75	3.47	1.27	6.51	0.012	1	0.062
	TA	4.89	1.85	4.72	1.44	0.25	0.616	1	0.003
	Econ	5.97	2.07	6.61	1.58	3.02	0.085	1	0.030
	SV	4.67	1.59	4.66	1.04	0.002	0.961	1	0.00003
	SA	4.40	1.48	4.35	1.37	0.04	0.852	1	0.00036
	EMot	6.28	2.12	6.48	1.58	0.29	0.594	1	0.003

*Note:* EA, empathic accuracy; Econ, emotional contagion; EMot, empathic motivation; HC, healthy control; PT, perspective taking; SA, self-emotional arousal; SCZ, schizophrenia; TV, target’s emotional valence; TA, target’s emotional arousal; SV, self-emotional valence.

#### EAT performance in negative-valenced videos

We found significant *main effects* of Group on the Empathic Accuracy, Perspective Taking, and Target’s Emotional Valence of cognitive empathy, with poorer performance in SCZ patients relative to healthy controls (see Table 2). Moreover, we found a significant *main effect* of Modality on the Emotional Contagion ratings of affective empathy, *F*(2, 196) = 8.54, *p* < 0.001, *η*_*p*_^2^ = 0.080. *Post hoc* comparisons revealed that Emotional Contagion ratings in audio-only (*M* = 6.66, *SD* = 2.09, Bonferroni-adjusted *p* < 0.001, Cohen’s *d* = 0.40) and audiovisual (*M* = 6.35, *SD* = 2.21, Bonferroni-adjusted *p* = 0.056, Cohen’s *d* = 0.24) conditions were significantly higher than in audioavatar visual condition (*M* = 5.87, *SD* = 2.19). We found a significant Modality-by-Group *interaction effect* for the Empathic Accuracy, *F*(2, 196) = 3.08, *p* = 0.048, *η*_*p*_^2^ = 0.031. As shown in Figure 1B, SCZ patients had lower EA scores than healthy controls in audio-only (Bonferroni-adjusted *p* = 0.002, Cohen’s *d* = −0.63) and audiovisual conditions (Bonferroni-adjusted *p* < 0.001, Cohen’s *d* = −0.83), but not in audioavatar visual condition (see Supplementary Table S1). No significant difference was found among three conditions within each group.

### HSoA–LSoA sample

Table 3 shows the demographics and self-reported measures of participants with HSoA and LSoA. The two groups were matched in demographics and estimated IQ. As expected, HSoA participants had higher physical and social anhedonia, but lower empathy and social functioning than LSoA participants.

**Table 3.** The characteristics of the HSoA–LSoA sample

	HSoA group (*n* = 59)		LSoA group (*n* = 60)		*t/χ^2^*	*p*	*df*	Cohen’s *d* [95% CI]
	*M*	*SD*	*M*	*SD*				
Sex (male:female)	28:31		30:30		0.08	0.781	1	
Age (year)	19.73	0.93	19.67	0.95	0.36	0.719	117	0.07 [−0.29, 0.43]
Years of education	13.61	1.22	13.92	0.96	−1.52	0.130	117	−0.28 [−0.64, 0.08]
CSAS	21.02	3.59	5.22	2.58	27.59	<0.001	117	5.06 [4.32, 5.80]
CPAS	18.22	8.37	9.72	6.15	6.30	<0.001	106.44	1.16 [0.77, 1.55]
QCAE_CE	57.73	7.27	61.25	6.83	−2.72	0.007	117	−0.50 [−0.86, −0.13]
QCAE_AE	29.02	5.28	30.97	4.80	−2.11	0.037	117	−0.39 [−0.75, −0.02]
SFS_total	74.20	6.86	81.98	7.47	−5.91	<0.001	117	−1.08 [−1.47, −0.70]

*Note:* AE, affective empathy; CE, cognitive empathy; CPAS, Chapman Physical Anhedonia Scale; CSAS, Chapman Social Anhedonia Scale; HSoA, high social anhedonia; LSoA, low social anhedonia; QCAE,  Cognitive and Affective Empathy Scale; SFS, First Episode Social Functioning Scale.

#### EAT performance in positive-valenced videos

We found significant *main effects* of Group for Empathic Accuracy and Target’s Emotional Valence within the domain of cognitive empathy, with the HSoA participants scoring significantly lower than the LSoA participants. We did not find any significant group difference in the remaining indices (see Table 4 and Figure 1C). The *main effect* of Modality on the Target’s Emotional Valence was significant, *F*(1.89, 221.02) = 4.78, *p* = 0.011, *η*_*p*_^2^ = 0.039. *Post hoc* comparison showed that the audiovisual condition (*M* = 7.79, *SD* = 1.04) was significantly higher than audio-only (*M* = 7.39, *SD* = 1.16; Bonferroni-adjusted *p* = 0.009, Cohen’s *d* = −0.28) and audioavatar visual (*M* = 7.45, *SD* = 1.27; Bonferroni-adjusted *p* = 0.027, Cohen’s *d* = −0.24) conditions. The significant *main effect* of Modality for the Target’s Emotional Arousal was observed, *F*(2, 234) = 3.84, *p* = 0.023, *η*_*p*_^2^ = 0.032. Audiovisual condition (*M* = 7.08, *SD* = 1.34) yielded significantly higher ratings than audio-only condition (*M* = 6.62, *SD* = 1.67; Bonferroni-adjusted *p* = 0.027, Cohen’s *d* = −0.25). Regarding affective empathy, we found significant *main effect* of Modality on the Self-emotional Valence, *F*(2, 234) = 8.94, *p* <0.001, *η*_*p*_^2^ = 0.071. The audiovisual condition (*M* = 6.83, *SD* = 1.17) was significantly higher than audio-only (*M* = 6.36, *SD* = 1.41; Bonferroni-adjusted *p* = 0.002, Cohen’s *d* = 0.32) and audioavatar visual (*M* = 6.34, *SD* = 1.32; Bonferroni-adjusted *p* <0.001, Cohen’s *d* = −0.37) conditions. Similarly, the *main effect* of Modality for the Self-emotional Arousal was significant, *F*(2, 234) = 8.56, *p* <0.001, *η*_*p*_^2^ = 0.068. Participants rated audiovisual condition (*M* = 6.08, *SD* = 1.76) higher than audio-only condition (*M* = 5.41, *SD* = 2.00; Bonferroni-adjusted *p* = 0.001, Cohen’s *d* = 0.35). However, we did not find any significant Modality-by-Group interaction in the HSoA–LSoA sample (see Supplementary Table S2).

**Table 4.** Group comparisons between participants with high and low social anhedonia on the EAT performance

		HSoA group(*n* = 59)		LSoA group(*n* = 60)		*F*	*p*	*df*	*η*_*p*_^2^
		*M*	*SD*	*M*	*SD*				
Positive videos	EA	0.87	0.29	1.05	0.27	12.28	0.001	1	0.095
	PT	7.15	1.35	7.40	1.02	1.33	0.251	1	0.011
	TV	7.40	0.80	7.69	0.71	4.47	0.037	1	0.037
	TA	6.66	1.09	7.03	1.02	3.57	0.061	1	0.030
	Econ	6.60	1.51	6.92	1.43	1.39	0.241	1	0.012
	SV	6.40	1.02	6.62	0.99	1.38	0.243	1	0.012
	SA	5.62	1.64	5.87	1.40	0.81	0.371	1	0.007
Negative videos	EA	0.77	0.38	0.88	0.39	2.53	0.115	1	0.021
	PT	6.37	1.40	6.95	1.22	5.71	0.018	1	0.047
	TV	3.54	1.50	3.12	1.13	2.98	0.087	1	0.025
	TA	4.90	1.45	5.01	1.40	0.17	0.682	1	0.001
	Econ	5.99	1.54	6.49	1.33	3.60	0.060	1	0.030
	SV	4.25	1.08	4.19	1.05	0.09	0.760	1	0.001
	SA	4.24	1.37	4.80	1.35	5.11	0.026	1	0.042
	EMot	6.04	1.39	6.96	1.42	12.85	<0.001	1	0.099

*Note:* EA, empathic accuracy; Econ, emotional contagion; EMot, empathic motivation; HSoA, high social anhedonia; LSoA, low social anhedonia; PT, perspective taking; SA, self-emotional arousal; SV, self-emotional valence; TV, target’s emotional valence; TA, target’s emotional arousal.

#### EAT performance in negative-valenced videos

We found significant *main effects* of Group in the ratings of Perspective Taking of cognitive empathy, Self-emotional Arousal of affective empathy, and Empathic Motivation, with HSoA participants scoring lower than LSoA participants (see Table 4). HSoA and LSoA participants did not differ in the remaining indices. However, the main effects of Modality and Modality-by-Group interaction effects were nonsignificant.

### Association between empathy deficit and clinical symptoms in SCZ patients

**Figure 2.**
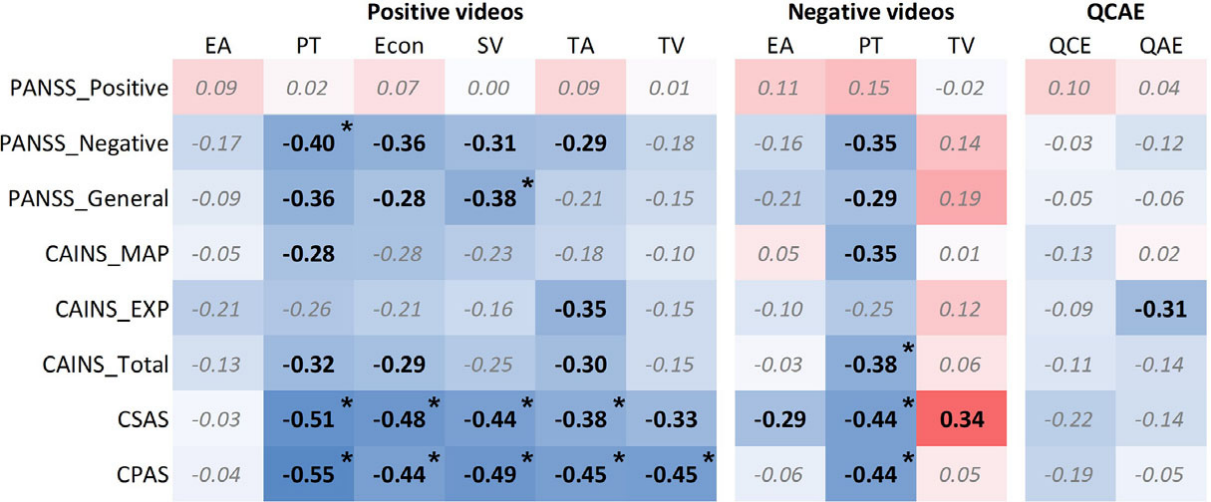
Association between empathy deficit and clinical symptoms in patients with schizophrenia. *Note*: CAINS, clinical assessment interview for negative symptoms; CPAS, Chapman Physical Anhedonia Scale; CSAS, Chapman Social Anhedonia Scale; EA, empathic accuracy; Econ, emotional contagion; EXP, expression; MAP, motivation and pleasure; PANSS, Positive and Negative Syndrome Scale; PT, perspective taking; QAE, affective empathy; QCAE, Cognitive and Affective Empathy Scale; QCE, cognitive empathy; SV, self-emotional valence; TA, target’s emotional arousal; TV, target’s emotional valence. *: FDR-adjusted *p* < 0.05.

In positive-valenced videos, Perspective Taking was negatively correlated with the PANSS negative factor (FDR-adjusted *p* = 0.032), the CSAS (FDR-adjusted *p* = 0.006) and the CPAS scores (FDR-adjusted *p* = 0.003). Emotional Contagion was also negatively correlated with the CSAS (FDR-adjusted *p* = 0.008) and CPAS (FDR-adjusted *p* = 0.012). Self-emotional Valence showed significant negative correlations with PANSS general symptoms (FDR-adjusted *p* = 0.045), the CSAS (FDR-adjusted *p* = 0.012) and CPAS scores (FDR-adjusted *p* = 0.008). Target’s Emotional Arousal was negatively correlated with the CSAS (FDR-adjusted *p* = 0.045), and CPAS scores (FDR-adjusted *p* = 0.012). Target’s Emotional Valence negatively correlated with the CPAS scores (FDR-adjusted *p* = 0.012). In negative-valenced videos, Perspective Taking was negatively correlated with the CAINS total score (FDR-adjusted *p* = 0.045) and the CSAS (FDR-adjusted *p* = 0.012), and CPAS scores (FDR-adjusted *p* = 0.012) (see Figure 2 and Supplementary Table S3). No significant correlations were found between dosage of antipsychotic medication and EAT performance.

## Discussion

Regardless of the emotional valence, we found significant Group main effect across the samples. Both SCZ patients and HSoA individuals showed lower cognitive empathy (especially Empathic Accuracy, Perspective Taking, Target’s Emotional Valence) relative to the respective comparison groups, which is consistent with previous research (Guo et al., 2023; Kamp et al., 2025; van Donkersgoed et al., 2019). Specifically, SCZ patients showed marked difficulties in understanding and inferring others’ mental states, regardless of positive or negative emotional valences. Such deficits appeared to be pervasive and trait-like features of SCZ patients, closely associated with social functioning (Green, Horan, & Lee, 2015). Similarly, HSoA individuals also exhibited cognitive empathy impairments, which may serve as an early indicator of SCZ spectrum disorders. Regarding affective empathy, SCZ patients exhibited deficits only in positive-valenced context (such as Emotional Contagion and Self-emotional Valence), while people with HSoA retained relatively intact affective empathy. Under the negative-valenced context, both SCZ patients and people with HSoA showed relatively intact affective empathy. This may indicate that SCZ patients have difficulty in processing of positive emotions, though they are able to experience and perceive others’ emotions in negative emotional stories. Such difficulty may stem from anhedonia and the weaker neural responses to social rewards (such as smiles and praise) (Guo et al., 2023; Lee et al., 2019), which adversely affect patients’ mental simulation abilities of others’ positive emotional states. In other words, SCZ patients may be unable to accurately map others’ happiness based on their own experiences, leading to a failure in affective empathy. Notably, our findings offer a new explanatory framework for previous inconsistent findings. The use of mixed emotional stimuli without distinguishing positive from negative emotional valence in previous research might have confounded prior results. After better delineating positive and negative emotion valences, affective empathy in SCZ patients was only found to be impaired in positive-valenced context. The Research Domain Criteria (RDoC) of the National Institute of Mental Health (NIMH), posits that dysfunction in the Positive Valence Systems is a core feature of SCZ (Cuthbert & Kozak, 2013; Insel et al., 2010). Our findings of SCZ patients having difficulty in processing rewards and positive stimuli concurred with the NIMH RDoC framework. Moreover, this study offers a new clinical direction for social cognitive interventions. The differential impairments in affective empathy implicate that targeted interventions for positive affective empathy can be more effective. For instance, positive emotion facial recognition training, and social reward scenario simulations can be applied in future research. Moreover, virtual reality technology can be used in the future to create positive social scenarios, enhancing patients’ experiences of others’ positive emotions, thereby improving their empathy abilities. In addition, HSoA individuals showed lower empathic motivation than people without social anhedonia, while no significant difference was observed between SCZ patients and controls. This may be due to the fact that we applied the extreme-group design to identify the HSoA and LSoA groups based on extremely-high scores on the CSAS, such as the CSAS ratings in HSoA participants were higher than SCZ participants (SCZ: HSoA = 13.7: 21.02) in this study. The HSoA group therefore expected less pleasure in social situations, resulting in social withdrawal (Mishlove & Chapman, 1985; Zhang et al., 2020).

Additionally, we found differential impacts of stimuli modalities by examining the Modality main effect and Group-by-Modality interaction. In the SCZ–Control sample, our findings indicated significant Modality main effect on both cognitive empathy (indexed by Target’s Emotional Valence) and affective empathy (indexed by Emotional Contagion). Specifically, participants exhibited poorer performance under audioavatar visual condition than audio-only and/or audiovisual conditions. Inaccurate facial expression information of Avatars may interfere with emotional recognition and empathic processing. In addition, we found significant Modality-by-Group interaction on Empathic Accuracy for both positive and negative valenced EAT. Under the positive-valenced context, visual information affected SCZ patients’ empathic processing, while under the negative-valenced context, SCZ patients had poorer empathic accuracy for audiovisual condition, but performed similarly to controls in interacting with the Avatar audiovideo condition. In the HSoA–LSoA sample, we found significant Modality main effect on both cognitive and affective empathy, with audiovisual condition outperformed than the other two conditions. However, the Modality types on empathy performance exhibited similar impacts to both groups with different levels of social anhedonia, which differed from the findings gathered in the SCZ–Control sample. In positive emotional events, individuals may exhibit more intense visual cues (e.g. clear facial expressions, dynamic gestures) (Zaki, Bolger, & Ochsner, 2009), while SCZ patients are impaired in the early stage of visual perception (Adámek, Langová, & Horáček, 2022; Matsumoto, Takahashi, Murai, & Takahashi, 2015), and there are significant deficits in audiovisual integration (Hirano, Nakamura, & Tamura, 2024; Lin, Ding, & Zhang, 2020). Furthermore, in these contexts, the visual information in audiovideo stimuli may increase the cognitive load for SCZ patients, acting as a distraction that interferes one’s ability to process auditory information. Consequently, both visual and auditory information are inadequately processed, impairing empathy accuracy in SCZ patients. However, differing from their counterparts with positive schizotypy, the visual perception of individuals with negative schizotypy such as social anhedonia may be preserved. This might be the reason why we did not find any significant interaction in HSoA–LSoA sample. Future research should include individuals with positive schizotypy.

The degree of expressiveness of video characters may influence empathy accuracy. For videos with less expressive targets, no significant difference in empathy accuracy was observed between SCZ patients and controls, while for more expressive targets, SCZ patients’ empathy accuracy was significantly lower (van Donkersgoed et al., 2019). Our findings confirmed these previous results in the context of negative emotional videos. Real-life emotion expressions can be sophisticated and complex, but Avatar technology can maximize characteristic features of emotion expression, thus reducing the complexity of emotional recognition. In negative emotional contexts, Avatar format may be simpler and easier for SCZ patients, and tax less on patients’ limited cognitive resources, thus improving empathy performance. Furthermore, interacting with Avatar virtual humans in a friendly environment may reduce social pressure for SCZ patients. Previous research has shown that children with autism perform better when interacting with virtual humans than with real humans (Pino, Vagnetti, Valenti, & Mazza, 2021). The nonjudgmental nature of virtual humans may also facilitate SCZ patients to focus more on perceiving the core emotional content, rather than inferring on others’ intention. Recent studies have combined virtual reality (VR) technology with social cognitive interaction training, and found this approach effective in improving SCZ patients’ social cognition (Cella et al., 2022; Shen et al., 2022).

Finally, the correlations analysis showed that performance of cognitive and affective empathy as measured by the EAT task in SCZ patients was inversely correlated with negative symptoms, consistent with previous research (Wang et al., 2021). SCZ patients having severe negative symptoms are believed to have greater difficulties in perceiving and inferring others’ emotional states. Given that diminished emotion expression is a feature of negative symptoms, it is plausible that SCZ patients having severe negative symptoms are unable to resonate with others’ emotions (Ma et al., 2024). Moreover, the CSAS and CPAS ratings in SCZ patients were inversely correlated with cognitive and affective empathy performance as measured using the EAT. The same patterns of correlations were also observed in the subclinical group (Wang et al., 2020b), which reported that cognitive empathy and affective empathy were negatively correlated with schizotypal traits, particularly social anhedonia. By integrating the findings from both samples, empathy deficits in SCZ spectrum disorder may be linked to negative symptoms.

Several limitations of our study should be borne in mind. First, we did not measure participants’ attention to the presented information, but variations in attention allocation could confound EAT task performance. Eye-tracking technology to explore gaze patterns while watching the videos can be used in future research, such that it is possible to analyze how visual information processing would affect empathy. Second, although we measured affective empathy and empathic motivation using postvideo ratings, these subjective ratings were prone to social desirability bias. Future research should incorporate more objective indicators (e.g. heart rate, skin conductance response, or other physiological data) to assess participants’ empathic responses. Third, we did not differentiate the clinical group into different subtypes of SCZ. The heterogeneity of our sample may affect the clinical validity of our findings. Fourth, our subclinical sample did not include people with high levels of positive schizotypal traits. Future studies should recruit larger samples, differentiate SCZ subtypes, and explore the empathic characteristics of clinical and subclinical groups.

In conclusion, both SCZ patients and HSoA individuals have impaired cognitive empathy, but cognitive empathy deficits in SCZ patients is influenced by the presentation modality and emotional valence. SCZ patients are impaired in affective empathy under positive-valenced context only, but HSoA individuals show relatively normal affective component of empathy.

## Supporting information

10.1017/S003329172610364X.sm001 Wang et al. supplementary material Wang et al. supplementary material

## Data Availability

The data that support the findings of this study are available from the corresponding author upon reasonable request.

## Supplementary material

The supplementary material for this article can be found at http://doi.org/10.1017/S003329172610364X.

## References

[r1] Adámek, P., Langová, V., & Horáček, J. (2022). Early-stage visual perception impairment in schizophrenia, bottom-up and back again. Schizophrenia, 8(1), 27. 10.1038/s41537-022-00237-9. 35314712 PMC8938488

[r2] American Psychiatric Association. (2013). Diagnostic and statistical manual of mental disorders. (5th ed.) Inc.: American Psychiatric Publishing.

[r3] Atoui, M., El Jamil, F., El Khoury, J., Doumit, M., Syriani, N., Khani, M., & Nahas, Z. (2018). The relationship between clinical insight and cognitive and affective empathy in schizophrenia. Schizophrenia Research: cognition, 12, 56–65. 10.1016/j.scog.2018.02.004. 29928598 PMC6007050

[r4] Baez, S., Herrera, E., Villarin, L., Theil, D., Gonzalez-Gadea, M. L., Gomez, P., … Ibañez, A. M. (2013). Contextual social cognition impairments in schizophrenia and bipolar disorder. PLoS One, 8(3), e57664. 10.1371/journal.pone.0057664. 23520477 PMC3592887

[r5] Barrantes-Vidal, N., Grant, P., & Kwapil, T. R. (2015). The role of schizotypy in the study of the etiology of schizophrenia spectrum disorders. Schizophrenia Bulletin, 41(suppl_2), S408 416. 10.1093/schbul/sbu191. 25810055 PMC4373635

[r6] Benedetti, F., Bernasconi, A., Bosia, M., Cavallaro, R., Dallaspezia, S., Falini, A., … Scotti, G. (2009). Functional and structural brain correlates of theory of mind and empathy deficits in schizophrenia. Schizophrenia Research, 114(1–3), 154–160. 10.1016/j.schres.2009.06.021. 19632816

[r7] Berger, P., Bitsch, F., Jakobi, B., Nagels, A., Straube, B., & Falkenberg, I. (2019). Cognitive and emotional empathy in patients with schizophrenia spectrum disorders: A replication and extension study. Psychiatry Research, 276, 56–59. 10.1016/j.psychres.2019.04.015. 31015067

[r8] Bertuccio, P., Amerio, A., Grande, E., La Vecchia, C., Costanza, A., Aguglia, A., & Odone, A. (2024). Global trends in youth suicide from 1990 to 2020: An analysis of data from the WHO mortality database. EClinical Medicine, 70, 102506. 10.1016/j.eclinm.2024.102506. PMC10911948 38440131

[r9] Bonfils, K. A., Lysaker, P. H., Minor, K. S., & Salyers, M. P. (2016). Affective empathy in schizophrenia: A meta-analysis. Schizophrenia Research, 175(1–3), 109–117. 10.1016/j.schres.2016.03.037. 27094715

[r10] Bonfils, K. A., Lysaker, P. H., Minor, K. S., & Salyers, M. P. (2017). Empathy in schizophrenia: A meta-analysis of the interpersonal reactivity index. Psychiatry Research, 249, 293–303. 10.1016/j.psychres.2016.12.033. 28142103

[r11] Boulic, R., Bécheiraz, P., Emering, L., & Thalmann, D. (1997). Integration of motion control techniques for virtual human and avatar real-time animation. In D. Thalmann, S. Feiner, & G. Singh (Eds.), Proceedings of the ACM symposium on virtual reality software and technology (pp. 111–118).ACM.

[r12] Cella, M., Tomlin, P., Robotham, D., Green, P., Griffiths, H., Stahl, D., & Valmaggia, L. (2022). Virtual reality therapy for the negative symptoms of schizophrenia (V-NeST): A pilot randomised feasibility trial. Schizophrenia Research, 248, 50–57. 10.1016/j.schres.2022.07.013. 35939920

[r13] Chan, R. C., Shi, C., Lui, S. S., Ho, K. K., Hung, K. S., Lam, J. W., … Yu, X. (2015). Validation of the Chinese version of the clinical assessment interview for negative symptoms (CAINS): A preliminary report. Frontiers in Psychology, 6, 7. 10.3389/fpsyg.2015.00007. 25698985 PMC4313598

[r14] Chapman, L. J., Chapman, J. P., & Raulin, M. L. (1976). Scales for physical and social anhedonia. Journal of Abnormal Psychology, 85(4), 374–382. 10.1037/0021-843X.85.4.374. 956504

[r15] Chu, X., Lu, X., & Huang, X. (2025). Does empathy predict bullying, or does bullying predict empathy? A meta-analysis of longitudinal studies. Journal of Youth and Adolescence. 10.1007/s10964-025-02223-7. 40699411

[r16] Couture, S. M., Penn, D. L., & Roberts, D. L. (2006). The functional significance of social cognition in schizophrenia: A review. Schizophrenia Bulletin, 32(suppl_1), S44–S63. 10.1093/schbul/sbl029. 16916889 PMC2632537

[r17] Cuthbert, B. N., & Kozak, M. J. (2013). Constructing constructs for psychopathology: The NIMH research domain criteria. Journal of Abnormal Psychology, 122(3), 928–937. 10.1037/a0034028. 24016027

[r18] de Jong, S., van Donkersgoed, R., Renard, S., Carter, S., Bokern, H., Lysaker, P., … Pijnenborg, G. H. M. (2018). Social-cognitive risk factors for violence in psychosis: A discriminant function analysis. Psychiatry Research, 265, 93–99. 10.1016/j.psychres.2018.04.048. 29702307

[r19] Derntl, B., Finkelmeyer, A., Toygar, T. K., Hülsmann, A., Schneider, F., Falkenberg, D. I., & Habel, U. (2009). Generalized deficit in all core components of empathy in schizophrenia. Schizophrenia Research, 108(1–3), 197–206. 10.1016/j.schres.2008.11.009. 19087898

[r20] Dominguez, M., Wichers, M., Lieb, R., Wittchen, H.-U., & van Os, J. (2011). Evidence that onset of clinical psychosis is an outcome of progressively more persistent subclinical psychotic experiences: An 8-year cohort study. Schizophrenia Bulletin, 37(1), 84–93. 10.1093/schbul/sbp022. 19460881 PMC3004179

[r21] Gesn, P. R., & Ickes, W. (1999). The development of meaning contexts for empathic accuracy: Channel and sequence effects. Journal of Personality and Social Psychology, 77(4), 746–761. 10.1037/0022-3514.77.4.746.

[r22] Giannitelli, M., Xavier, J., François, A., Bodeau, N., Laurent, C., Cohen, D., & Chaby, L. (2015). Facial, vocal and cross-modal emotion processing in early-onset schizophrenia spectrum disorders. Schizophrenia Research, 168(1–2), 252–259. 10.1016/j.schres.2015.07.039. 26297473

[r23] Gong, Y.-x., & Dai, X.-y. (1984). The short-form application of the Wechsler intelligence scale. Journal of Hunan Medical College, (04), 393–401.

[r24] Green, M. F., Horan, W. P., & Lee, J. (2015). Social cognition in schizophrenia. Nature Reviews Neuroscience, 16(10), 620–631. 10.1038/nrn4005. 26373471

[r25] Gu, J., Ding, X., Yang, J., Meng, X., Hu, W., Li, X., … Chan, R. C. K. (2025). Individuals with high social anhedonia but not schizophrenia exhibited altered empathy in daily life. Schizophrenia Bulletin, sbaf136. 10.1093/schbul/sbaf136. 40825191 PMC13391632

[r26] Guo, X. d., Zheng, H., Ruan, D., Wang, Y., Wang, Y. y., & Chan, R. C. (2023). Altered empathy correlates with reduced social and non-social reward anticipation in individuals with high social anhedonia. PsyCh Journal, 12(1), 92–99. 10.1002/pchj.592. 36058882

[r27] Hall, J. A., & Schmid Mast, M. (2007). Sources of accuracy in the empathic accuracy paradigm. Emotion, 7(2), 438–446. 10.1037/1528-3542.7.2.438. 17516820

[r28] Halverson, T. F., Orleans-Pobee, M., Merritt, C., Sheeran, P., Fett, A. K., & Penn, D. L. (2019). Pathways to functional outcomes in schizophrenia spectrum disorders: Meta-analysis of social cognitive and neurocognitive predictors. Neuroscience & Biobehavioral Reviews, 105, 212–219. 10.1016/j.neubiorev.2019.07.020. 31415864

[r29] Harenski, C. L., Brook, M., Kosson, D. S., Bustillo, J. R., Harenski, K. A., Caldwell, M. F., … Kiehl, K. A. (2017). Socio-neuro risk factors for suicidal behavior in criminal offenders with psychotic disorders. Social Cognitive and Affective Neuroscience, 12(1), 70–80. 10.1093/scan/nsw164. 28065894 PMC5390707

[r30] Harvey, P.-O., Zaki, J., Lee, J., Ochsner, K., & Green, M. F. (2013). Neural substrates of empathic accuracy in people with schizophrenia. Schizophrenia Bulletin, 39(3), 617–628. 10.1093/schbul/sbs042. 22451493 PMC3627780

[r31] Hirano, Y., Nakamura, I., & Tamura, S. (2024). Abnormal connectivity and activation during audiovisual speech perception in schizophrenia. The European Journal of Neuroscience, 59(8), 1918–1932. 10.1111/ejn.16183. 37990611

[r32] Hu, D., Guo, X., Zheng, H., Yan, C., Lui, S. S. Y., Wang, Y., … Chan, R. C. K. (2024). Empathic accuracy in individuals with schizotypal personality traits. PsyCh Journal, 13(5), 813–823. 10.1002/pchj.743. 38530878 PMC11444733

[r33] Insel, T., Cuthbert, B., Garvey, M., Heinssen, R., Pine, D. S., Quinn, K., … Wang, P. (2010). Research domain criteria (RDoC): Toward a new classification framework for research on mental disorders. American Journal of Psychiatry, 167(7), 748–751. 10.1176/appi.ajp.2010.09091379. 20595427

[r34] Jospe, K., Genzer, S., Selle, N., Ong, D., Zaki, J., & Perry, A. (2020). The contribution of linguistic and visual cues to physiological synchrony and empathic accuracy. Cortex, 132, 296–308. 10.1016/j.cortex.2020.09.001. 33010739

[r35] Kamp, D., Hartmann, N., Frommann, N., Lowe, A., Pintgen, L., Weide, K., & Wölwer, W. (2025). The relationship between empathy, theory of mind and facial affect recognition in schizophrenia patients.*The* International Journal of Neuroscience, 135(7), 741–745. 10.1080/00207454.2024.2327415. 38441493

[r36] Karpouzian-Rogers, T., Cobia, D., Petersen, J., Wang, L., Mittal, V. A., Csernansky, J. G., & Smith, M. J. (2021). Cognitive empathy and longitudinal changes in Temporo-parietal junction thickness in schizophrenia. Frontiers in Psychiatry, 12, 667656. 10.3389/fpsyt.2021.667656. 34054621 PMC8160364

[r37] Kay, S. R., Fiszbein, A., & Opler, L. A. (1987). The positive and negative syndrome scale (PANSS) for schizophrenia. Schizophrenia Bulletin, 13(2), 261–276. 10.1093/schbul/13.2.261. 3616518

[r38] Knobloch, S., Leiding, D., Wagels, L., Regenbogen, C., Kellermann, T., Mathiak, K., … Habel, U. (2024). Empathy in schizophrenia: Neural alterations during emotion recognition and affective sharing. Frontiers in Psychiatry, 15, 1288028. 10.3389/fpsyt.2024.1288028. 38855645 PMC11157094

[r39] Kraus, M. W. (2017). Voice-only communication enhances empathic accuracy. American Psychologist, 72(7), 644–654. 10.1037/amp0000147. 29016168

[r40] Kring, A. M., Gur, R. E., Blanchard, J. J., Horan, W. P., & Reise, S. P. (2013). The clinical assessment interview for negative symptoms (CAINS): Final development and validation. American Journal of Psychiatry, 170(2), 165–172. 10.1176/appi.ajp.2012.12010109. 23377637 PMC3785242

[r41] Kwapil, T. R. (1998). Social anhedonia as a predictor of the development of schizophrenia-spectrum disorders. Journal of Abnormal Psychology, 107(4), 558–565. 10.1037/0021-843x.107.4.558. 9830243

[r42] Lecomte, T., Corbière, M., Ehmann, T., Addington, J., Abdel-Baki, A., & Macewan, B. (2014). Development and preliminary validation of the first episode social functioning scale for early psychosis. Psychiatry Research, 216(3), 412–417. 10.1016/j.psychres.2014.01.044. 24613006

[r43] Lecrubier, Y., Sheehan, D. V., Weiller, E., Amorim, P., Bonora, I., Sheehan, K. H., … Dunbar, G. C. (1997). The MINI international neuropsychiatric interview (MINI). A short diagnostic structured interview: Reliability and validity according to the CIDI. European Psychiatry, 12(5), 224–231. 10.1016/S0924-9338(97)83296-8.

[r44] Lee, J., Jimenez, A. M., Reavis, E. A., Horan, W. P., Wynn, J. K., & Green, M. F. (2019). Reduced neural sensitivity to social vs nonsocial reward in schizophrenia. Schizophrenia Bulletin, 45(3), 620–628. 10.1093/schbul/sby109. 30189096 PMC6483569

[r45] Lee, J., Zaki, J., Harvey, P.-O., Ochsner, K., & Green, M. (2011). Schizophrenia patients are impaired in empathic accuracy. Psychological Medicine, 41(11), 2297–2304. 10.1017/S0033291711000614. 21524334 PMC3928128

[r46] Lee, S. J., Kang, D. H., Kim, C. W., Gu, B. M., Park, J. Y., Choi, C. H., … Kwon, J. S. (2010). Multi-level comparison of empathy in schizophrenia: An fMRI study of a cartoon task. Psychiatry Research, 181(2), 121–129. 10.1016/j.pscychresns.2009.08.003. 20080395

[r47] Liang, Y. s., Yang, H. x., Ma, Y. t., Lui, S. S., Cheung, E. F., Wang, Y., & Chan, R. C. (2019). Validation and extension of the questionnaire of cognitive and affective empathy in the Chinese setting. PsyCh Journal, 8(4), 439–448. 10.1002/pchj.281. 30983167

[r48] Lin, Y., Ding, H., & Zhang, Y. (2020). Multisensory integration of emotion in schizophrenic patients. Multisensory Research, 33(8), 865–901. 10.1163/22134808-bja10016. 33706267

[r49] Ma, Z., Tian, Y., Li, J., Liu, J., Wang, D.-M., & Zhang, X.-Y. (2024). Association of empathy with clinical symptoms and cognitive function in chronic schizophrenia patients with and without suicide attempts. European Archives of Psychiatry and Clinical Neuroscience, 274(6), 1395–1404. 10.1007/s00406-024-01785-0. 38478155

[r50] Matsumoto, Y., Takahashi, H., Murai, T., & Takahashi, H. (2015). Visual processing and social cognition in schizophrenia: Relationships among eye movements, biological motion perception, and empathy. Neuroscience Research, 90, 95–100. 10.1016/j.neures.2014.10.011. 25449145

[r51] Meehl, P. E. (1962). Schizotaxia, schizotypy, schizophrenia. American Psychologist, 17(12), 827–838. 10.1037/h0041029.

[r52] Mishlove, M., & Chapman, L. J. (1985). Social anhedonia in the prediction of psychosis proneness. Journal of Abnormal Psychology, 94(3), 384–396. 10.1037/0021-843x.94.3.384. 4031235

[r53] Ong, D. C., Jospe, K., Reddan, M., Wu, Z., Kahhale, I., Chen, P., & Perry, A. (2023). People optimally and flexibly process emotional information across multiple modalities. PsyArXiv. 10.31234/osf.io/5pr6w.

[r54] Paulmann, S., & Pell, M. D. (2011). Is there an advantage for recognizing multi-modal emotional stimuli? Motivation and Emotion, 35(2), 192–201. 10.1007/s11031-011-9206-0.

[r55] Pflum, M. J., & Gooding, D. C. (2018). Context matters: Social cognition task performance in psychometric schizotypes. Psychiatry Research, 264, 398–403. 10.1016/j.psychres.2018.03.075. 29679842

[r56] Pino, M. C., Vagnetti, R., Valenti, M., & Mazza, M. (2021). Comparing virtual vs real faces expressing emotions in children with autism: An eye-tracking study. Education and Information Technologies, 26(5), 5717–5732. 10.1007/s10639-021-10552-w.

[r57] Preston, S. D., & de Waal, F. B. (2002). Empathy: Its ultimate and proximate bases. The Behavioral and Brain Sciences, 25(1), 1–71. 10.1017/s0140525x02000018. 12625087

[r58] Ramos-Loyo, J., Mora-Reynoso, L., Sánchez-Loyo, L. M., & Medina-Hernández, V. (2012). Sex differences in facial, prosodic, and social context emotional recognition in early-onset schizophrenia. Schizophrenia Research and Treatment, 2012(1), 584725. 10.1155/2012/584725. 22970365 PMC3420677

[r59] Reniers, R. L., Corcoran, R., Drake, R., Shryane, N. M., & Völlm, B. A. (2011). The QCAE: A questionnaire of cognitive and affective empathy. Journal of Personality Assessment, 93(1), 84–95. 10.1080/00223891.2010.528484. 21184334

[r60] Serafini, G., Aguglia, A., Amerio, A., Canepa, G., Adavastro, G., Conigliaro, C., … Amore, M. (2023). The relationship between bullying victimization and perpetration and non-suicidal self-injury: A systematic review. Child Psychiatry and Human Development, 54(1), 154–175. 10.1007/s10578-021-01231-5. 34435243 PMC9867675

[r61] Shen, Z.-H., Liu, M.-H., Wu, Y., Lin, Q.-Q., & Wang, Y.-G. (2022). Virtual-reality-based social cognition and interaction training for patients with schizophrenia: A preliminary efficacy study. Frontiers in Psychiatry, 13, 1022278. 10.3389/fpsyt.2022.1022278. 36465308 PMC9714325

[r62] Si, T. -m., Yang, J. -z., Shu-Liang , Wang, X. -l., Kong, Q. m., Zhou-Mo , … Liu-cui . (2004). Reliability and validity of the positive and negative syndrome scale (PANSS, Chinese version). Chinese Mental Health Journal, 18(01), 45–47.

[r63] Simpson, C., Pinkham, A. E., Kelsven, S., & Sasson, N. J. (2013). Emotion recognition abilities across stimulus modalities in schizophrenia and the role of visual attention. Schizophrenia Research, 151(1–3), 102–106. 10.1016/j.schres.2013.09.026. 24126043

[r64] Smith, M. J., Horan, W. P., Cobia, D. J., Karpouzian, T. M., Fox, J. M., Reilly, J. L., & Breiter, H. C. (2014). Performance-based empathy mediates the influence of working memory on social competence in schizophrenia. Schizophrenia Bulletin, 40(4), 824–834. 10.1093/schbul/sbt084. 23770935 PMC4059427

[r65] Sung, E. C., Han, D.-I. D., Bae, S., & Kwon, O. (2022). What drives technology-enhanced storytelling immersion? The role of digital humans. Computers in Human Behavior, 132, 107246. 10.1016/j.chb.2022.107246.

[r66] Thaler, N. S., Strauss, G. P., Sutton, G. P., Vertinski, M., Ringdahl, E. N., Snyder, J. S., & Allen, D. N. (2013). Emotion perception abnormalities across sensory modalities in bipolar disorder with psychotic features and schizophrenia. Schizophrenia Research, 147(2–3), 287–292. 10.1016/j.schres.2013.04.001. 23611243

[r67] van Donkersgoed, R. J. M., de Jong, S., Aan Het Rot, M., Wunderink, L., Lysaker, P. H., Hasson-Ohayon, I., … Pijnenborg, G. H. M. (2019). Measuring empathy in schizophrenia: The empathic accuracy task and its correlation with other empathy measures. Schizophrenia Research, 208, 153–159. 10.1016/j.schres.2019.03.024. 31006615

[r68] van Noorden, T. H. J., Haselager, G. J. T., Cillessen, A. H. N., & Bukowski, W. M. (2015). Empathy and involvement in bullying in children and adolescents: A systematic review. Journal of Youth and Adolescence, 44(3), 637–657. 10.1007/s10964-014-0135-6. 24894581

[r69] Vaskinn, A., Andersson, S., Østefjells, T., Andreassen, O. A., & Sundet, K. (2018). Emotion perception, non-social cognition and symptoms as predictors of theory of mind in schizophrenia. Comprehensive Psychiatry, 85, 1–7. 10.1016/j.comppsych.2018.05.002. 29906670

[r70] Vogel, B. D., Brück, C., Jacob, H., Eberle, M., & Wildgruber, D. (2016). Effects of cue modality and emotional category on recognition of nonverbal emotional signals in schizophrenia. BMC Psychiatry, 16(1), 218. 10.1186/s12888-016-0913-7. 27388011 PMC4936116

[r71] Wang, L., Lui, S. S. Y., & Chan, R. C. K. (2024). Neuropsychology and neurobiology of negative schizotypy: A selective review. Biological Psychiatry Global Open Science, 4(4), 100317. 10.1016/j.bpsgos.2024.100317. 38711865 PMC11070600

[r72] Wang, M., Zhang, L., Fu, X., Wang, Y., Jiang, Y., Cao, Y., … Raymond, C. K. C. (2025). Investigating the differential effects of audio-visual information and emotional valence on empathic accuracy. Journal of Psychological Science, 48(3), 567–576. 10.16719/j.cnki.1671-6981.20250306.

[r73] Wang, W., Zhou, Y., Liu, R., Wei, S., Xu, H., Wang, J., … Wang, D. (2021). Association between empathy and clinical symptoms in chronic schizophrenia: A large sample study based on Chinese Han population. Journal of Psychiatric Research, 139, 106–112. 10.1016/j.jpsychires.2021.05.046. 34058648

[r74] Wang, W., Zhou, Y., Wang, J., Xu, H., Wei, S., Wang, D., … Zhang, X. Y. (2020a). Prevalence, clinical correlates of suicide attempt and its relationship with empathy in patients with schizophrenia. Progress in Neuro-Psychopharmacology & Biological Psychiatry, 99, 109863. 10.1016/j.pnpbp.2020.109863. 31931089

[r75] Wang, Y., Shi, H.-s., Liu, W.-h., Zheng, H., Wong, K. K.-Y., Cheung, E. F., & Chan, R. C. (2020b). Applying network analysis to investigate the links between dimensional schizotypy and cognitive and affective empathy. Journal of Affective Disorders, 277, 313–321. 10.1016/j.jad.2020.08.030. 32858312

[r76] Wang, Y., Yeh, Y.-h., Tsang, S.-m., Liu, W.-h., Shi, H.-s., Li, Z., … Lui, S. S. (2013). Social functioning in Chinese college students with and without schizotypal personality traits: An exploratory study of the Chinese version of the first episode social functioning scale. PLoS One, 8(5), e61115. 10.1371/journal.pone.0061115. 23690922 PMC3653910

[r77] Zaki, J., Bolger, N., & Ochsner, K. (2009). Unpacking the informational bases of empathic accuracy. Emotion, 9(4), 478–487. 10.1037/a0016551. 19653768 PMC6558959

[r78] Zhang, H., Chen, X., Chen, S., Li, Y., Chen, C., Long, Q., & Yuan, J. (2018). Facial expression enhances emotion perception compared to vocal prosody: Behavioral and fMRI studies. Neuroscience Bulletin, 34(5), 801–815. 10.1007/s12264-018-0231-9. 29740753 PMC6129238

[r79] Zhang, R. -t., Yang, Z. -y., Wang, Y. -m., Wang, Y., Yang, T. -X., Cheung, E. F., … Chan, R. C. (2020). Affective forecasting in individuals with social anhedonia: The role of social components in anticipated emotion, prospection and neural activation. Schizophrenia Research, 215, 322–329. 10.1016/j.schres.2019.10.006. 31611042

