# Supplementary material for: Differential impacts of audiovisual information on empathic accuracy in people with schizophrenia and high social anhedonia
Source: Psychol Med. 2026 Mar 23;56:e72. doi: 10.1017/S003329172610364X (PMC13079236; doi:10.1017/S003329172610364X)
Supplement: Wang et al. supplementary material [file S003329172610364Xsup001.docx]

**Supplementary Materials**

**Table S1 Results of the Interaction Between Modality-Condition and Group in SCZ-Control Sample**

|  |  | SCZ group (n=50) | | |  | HC group (n=50) | | |  |  |  |  |
| --- | --- | --- | --- | --- | --- | --- | --- | --- | --- | --- | --- | --- |
|  |  | AO | A-AV | AV |  | AO | A-AV | AV | *F* | *p* | *df* | *η_p_^2^* |
| Positive videos | EA | 0.71±0.66 | 0.47±0.74 | 0.67±0.71 |  | 0.83±0.46 | 1.01±0.37 | 0.93±0.50 | 5.61 | **0.004** | 2 | 0.054 |
|  | PT | 6.48±2.03 | 5.94±2.27 | 6.48±2.22 |  | 7.48±1.37 | 7.42±1.67 | 7.36±1.68 | 1.63 | 0.199 | 2 | 0.016 |
|  | TV | 6.40±2.19 | 5.86±2.47 | 6.50±2.43 |  | 7.70±1.30 | 7.58±1.16 | 7.92±1.10 | 0.64 | 0.527 | 2 | 0.007 |
|  | TA | 5.84±2.32 | 6.02±2.30 | 5.98±2.71 |  | 7.12±1.24 | 7.06±1.53 | 6.96±1.60 | 0.24 | 0.761 | 1.77 | 0.002 |
|  | Econ | 6.28±2.05 | 5.72±2.23 | 6.28±2.18 |  | 6.86±1.57 | 6.90±1.89 | 7.20±1.70 | 1.08 | 0.337 | 1.78 | 0.011 |
|  | SV | 5.76±2.08 | 5.40±2.02 | 5.84±2.12 |  | 6.28±1.41 | 6.52±1.37 | 6.48±1.61 | 1.27 | 0.282 | 1.86 | 0.013 |
|  | SA | 5.40±2.05 | 5.52±2.04 | 5.90±2.17 |  | 5.62±1.84 | 5.90±1.76 | 5.80±1.80 | 0.68 | 0.506 | 2 | 0.007 |
| Negative videos | EA | 0.14±0.90 | 0.26±0.78 | 0.08±0.85 |  | 0.61±0.57 | 0.52±0.68 | 0.71±0.67 | 3.08 | **0.048** | 2 | 0.031 |
|  | PT | 6.06±2.39 | 5.64±2.24 | 5.64±2.27 |  | 6.96±1.83 | 6.74±1.90 | 6.52±2.00 | 0.16 | 0.855 | 2 | 0.002 |
|  | TV | 4.24±2.23 | 3.92±2.09 | 4.58±2.14 |  | 3.66±1.84 | 3.30±1.74 | 3.44±1.69 | 1.07 | 0.346 | 2 | 0.011 |
|  | TA | 4.80±2.45 | 4.88±2.30 | 4.98±2.43 |  | 4.76±2.03 | 4.54±1.66 | 4.86±2.00 | 0.21 | 0.811 | 2 | 0.002 |
|  | Econ | 6.40±2.36 | 5.50±2.27 | 6.02±2.30 |  | 6.92±1.76 | 6.24±2.06 | 6.68±2.09 | 0.17 | 0.846 | 2 | 0.002 |
|  | SV | 4.50±1.96 | 4.46±2.02 | 5.06±2.00 |  | 4.80±1.39 | 4.56±1.47 | 4.62±1.40 | 2.05 | 0.132 | 2 | 0.020 |
|  | SA | 4.26±2.10 | 4.70±2.15 | 4.24±2.37 |  | 4.40±1.76 | 4.24±1.80 | 4.40±1.84 | 1.02 | 0.358 | 1.86 | 0.010 |
|  | EM | 6.58±2.20 | 5.91±2.35 | 6.34±2.41 |  | 6.47±1.81 | 6.55±1.72 | 6.41±2.05 | 2.72 | 0.068 | 2 | 0.027 |

**Table S2 Results of the Interaction Between Modality-Condition and Group in HSoA-LSoA Sample**

|  |  | HSoA group (n=59) | | |  | LSoA group (n=60) | | |  |  |  |  |
| --- | --- | --- | --- | --- | --- | --- | --- | --- | --- | --- | --- | --- |
|  |  | AO | A-AV | AV |  | AO | A-AV | AV | *F* | *p* | *df* | *η_p_^2^* |
| Positive videos | EA | 0.91±0.49 | 0.86±0.49 | 0.84±0.47 |  | 1.07±0.34 | 1.08±0.45 | 1.01±0.41 | 0.20 | 0.820 | 2 | 0.002 |
|  | PT | 7.15±1.53 | 6.90±1.99 | 7.39±1.33 |  | 7.23±1.43 | 7.43±1.33 | 7.53±1.38 | 1.40 | 0.250 | 2 | 0.012 |
|  | TV | 7.29±1.29 | 7.31±1.41 | 7.59±1.13 |  | 7.50±1.02 | 7.58±1.12 | 7.98±0.91 | 0.21 | 0.798 | 1.89 | 0.002 |
|  | TA | 6.49±1.78 | 6.66±1.63 | 6.83±1.23 |  | 6.75±1.56 | 7.00±1.29 | 7.33±1.40 | 0.28 | 0.757 | 2 | 0.002 |
|  | Econ | 6.69±1.82 | 6.49±2.20 | 6.63±1.81 |  | 6.57±1.81 | 7.00±1.66 | 7.20±1.71 | 2.43 | 0.091 | 2 | 0.020 |
|  | SV | 6.27±1.48 | 6.34±1.35 | 6.59±1.21 |  | 6.45±1.33 | 6.33±1.30 | 7.07±1.10 | 1.69 | 0.188 | 2 | 0.014 |
|  | SA | 5.44±1.99 | 5.59±1.92 | 5.83±1.95 |  | 5.38±2.03 | 5.90±1.50 | 6.33±1.54 | 1.54 | 0.216 | 2 | 0.013 |
| Negative videos | EA | 0.76±0.52 | 0.74±0.54 | 0.80±0.54 |  | 0.93±0.50 | 0.88±0.58 | 0.84±0.57 | 0.71 | 0.493 | 2 | 0.006 |
|  | PT | 6.51±2.02 | 6.49±2.17 | 6.12±2.04 |  | 6.78±1.75 | 7.00±1.73 | 7.07±1.51 | 1.29 | 0.279 | 2 | 0.011 |
|  | TV | 3.54±1.79 | 3.53±1.81 | 3.56±1.82 |  | 3.00±1.38 | 3.12±1.64 | 3.25±1.34 | 0.29 | 0.748 | 2 | 0.002 |
|  | TA | 4.69±1.92 | 5.14±1.91 | 4.88±1.95 |  | 4.73±1.96 | 5.23±1.72 | 5.07±1.90 | 0.07 | 0.933 | 2 | 0.001 |
|  | Econ | 6.12±2.20 | 6.15±2.03 | 5.71±2.30 |  | 6.43±1.87 | 6.40±1.92 | 6.65±1.64 | 1.47 | 0.232 | 2 | 0.012 |
|  | SV | 4.31±1.50 | 4.34±1.29 | 4.12±1.47 |  | 4.23±1.29 | 4.15±1.45 | 4.20±1.36 | 0.45 | 0.641 | 2 | 0.004 |
|  | SA | 4.17±1.88 | 4.20±1.83 | 4.34±1.88 |  | 4.55±1.69 | 5.00±1.89 | 4.85±1.67 | 0.63 | 0.532 | 2 | 0.005 |
|  | EM | 6.18±1.77 | 5.85±2.01 | 6.10±1.85 |  | 6.98±1.63 | 6.87±1.89 | 7.04±1.57 | 0.18 | 0.832 | 2 | 0.002 |

**Table S3** Correlation Between Empathy Indices and Clinical Symptoms in Schizophrenia Patients

|  | Positive videos | | | | | |  | Negative videos | | |  |  |
| --- | --- | --- | --- | --- | --- | --- | --- | --- | --- | --- | --- | --- |
|  | EA | PT | Econ | SV | TA | TV |  | EA | PT | TV | QCAE_QCE | QCAE_QAE |
| PANSS_P | 0.090 | 0.017 | 0.068 | -0.004 | 0.087 | 0.008 |  | 0.111 | 0.147 | -0.016 | 0.097 | 0.040 |
| PANSS_N | -0.169 | -0.400^**^ | -0.363^**^ | -0.313^*^ | -0.292^*^ | -0.179 |  | -0.160 | -0.350^*^ | 0.140 | -0.031 | -0.120 |
| PANSS_G | -0.086 | -0.357^*^ | -0.279^*^ | -0.375^**^ | -0.208 | -0.152 |  | -0.210 | -0.290^*^ | 0.192 | -0.049 | -0.055 |
| CAINS_MAP | -0.051 | -0.279^*^ | -0.275 | -0.231 | -0.180 | -0.100 |  | 0.050 | -0.352^*^ | 0.007 | -0.125 | 0.020 |
| CAINS_EXP | -0.210 | -0.258 | -0.212 | -0.164 | -0.350^*^ | -0.151 |  | -0.103 | -0.246 | 0.121 | -0.094 | -0.313^*^ |
| CAINS_Total | -0.132 | -0.319^*^ | -0.291^*^ | -0.247 | -0.301^*^ | -0.153 |  | -0.027 | -0.376^**^ | 0.056 | -0.109 | -0.139 |
| CSAS | -0.028 | -0.514^**^ | -0.483^**^ | -0.443^**^ | -0.380^**^ | -0.327^*^ |  | -0.290^*^ | -0.444^**^ | 0.340^*^ | -0.218 | -0.144 |
| CPAS | -0.044 | -0.553^**^ | -0.443^**^ | -0.486^**^ | -0.447^**^ | -0.453^**^ |  | -0.062 | -0.442^**^ | 0.045 | -0.193 | -0.046 |

Note: P = Positive symptom；N = Negative symptoms; G = General pathological symptoms; MAP = Motivation and Pleasure Dimensions; EXP = Expression dimension; T = Total; **p* < 0.05；***p* < 0.01；****p* < 0.001
